# The enhanced recovery programme pilot: can we achieve better outcomes and shorter stays for cardiac surgical patients?

**DOI:** 10.1186/1749-8090-8-S1-O1

**Published:** 2013-09-11

**Authors:** Louise Kenny, Thasee Pillay, David Kinnersley

**Affiliations:** 1 Cardiothoracic Directorate, Freeman Hospital, Newcastle, UK

## Background

Enhanced recovery programmes (ERP) reduce hospital stay and improve outcomes through a structured approach to perioperative care. We present our tailored protocol and results for first time AVR and CABG patients enrolled on ERP. Key elements are optimising co-morbidities, discharge planning, reduced starvation with carbohydrate loading. Intra-op focus is on minimally invasive techniques and analgesic planning. Post-op protocol aims to extubate early, reduce opiate analgesia using other agents, rapid mobilisation and return to normal diet and function.

## Methods

27 CABG and 13 AVR patients were entered onto ERP. Combined procedures and those with complex social requirements were excluded. Length of ICU and hospital stay (LOS), complications, readmissions and mortality were evaluated.

## Results

Mean age was 67.5 (CABG) and 72.9 (AVR). ICU LOS ranged 0.7-5.1days (median 0.9) for CABG and 0.7-18.8 days (median 0.9) for AVR. Hospital stay ranged 3-8 days (median 5) for CABG and 5-35 days (median 9) for AVR. Readmission rates were 3.7% for CABG (n=1 readmitted day 34 postop with a wound infection) and 15.1% for AVR (n=2 with 1 patient readmitted at day 5 due to wound infection and 1 at day 60 with endocarditis). Overall 30 day readmission rate=2.5%. No deaths or major complications occurred in the CABG group.

In the AVR group, 1 death occurred 65 days post op due to endocarditis, 1 patient had a protracted ICU stay due to respiratory failure, 2 patients required wound debridement. No patient in either group required renal replacement therapy. In our unit median ICU stay is 1 day and inpatient stay is 6 days for CABG. Our results show 17% reduction in LOS for CABG patients.

## Conclusion

ERP clearly reduces LOS for selected elective cardiac procedures compared to non-ERP patients and ICU stays are shorter. The reduced LOS, and therefore increased capacity associated with ERP has implications on patient experience and valuable hospital resources.

